# Hippocampal protein kinase D1 is necessary for DHPG-induced learning and memory impairments in rats

**DOI:** 10.1371/journal.pone.0195095

**Published:** 2018-04-03

**Authors:** Wei Wang, Florian Duclot, Bradley R. Groveman, Nicole Carrier, Haifa Qiao, Xiao-Qian Fang, Hui Wang, Wenkuan Xin, Xing-Hong Jiang, Michael W. Salter, Xin-Sheng Ding, Mohamed Kabbaj, Xian-Min Yu

**Affiliations:** 1 Department of Neurology, the First Affiliated Hospital of Nanjing Medical University, Nanjing, People’s Republic of China; 2 BenQ Affiliated Hospital and Neurological Institute, Nanjing Medical University, Nanjing, People’s Republic of China; 3 Department of Biomedical Sciences, Florida State University, Tallahassee, Florida, United States of America; 4 Department of Biomedical Sciences, University of Texas Rio Grande Valley School of Medicine, Edinburg, Texas, United States of America; 5 College of Pharmaceutical Sciences, Southwest University, Chongqing, People’s Republic of China; 6 Department of Physiology and Neurobiology, Medical College of Soochow University, Suzhou, People’s Republic of China; 7 Program in Neuroscience and Mental Health, Hospital for Sick Children, University of Toronto, Toronto, Ontario, Canada; University of Louisville, UNITED STATES

## Abstract

**Background:**

Understanding molecular mechanisms underlying the induction of learning and memory impairments remains a challenge. Recent investigations have shown that the activation of group I mGluRs (mGluR1 and mGluR5) in cultured hippocampal neurons by application of (*S*)-3,5-Dihydroxyphenylglycine (DHPG) causes the regulated internalization of N-methyl-D-aspartate receptors (NMDARs), which subsequently activates protein kinase D1 (PKD1). Through phosphorylating the C-terminals of the NMDAR GluN2 subunits, PKD1 down-regulates the activity of remaining (non-internalized) surface NMDARs. The knockdown of PKD1 does not affect the DHPG-induced inhibition of AMPA receptor-mediated miniature excitatory post-synaptic currents (mEPSCs) but prevents the DHPG-induced inhibition of NMDAR-mediated mEPSCs *in vitro*. Thus, we investigated the *in vivo* effects of bilateral infusions of DHPG into the hippocampal CA1 area of rats in the Morris water maze (MWM) and the novel object discrimination (NOD) tests.

**Methods:**

A total of 300 adult male Sprague Dawley rats (250–280 g) were used for behavioral tests. One hundred ninety four were used in MWM test and the other 106 rats in the NOD test. Following one week of habituation to the vivarium, rats were bilaterally implanted under deep anesthesia with cannulas aimed at the CA1 area of the hippocampus (CA1 coordinates in mm from Bregma: AP -3.14; lateral +/-2; DV -3.0). Through implanted cannulas artificial cerebrospinal fluid (ACSF), the group1 mGluR antagonist 6-Methyl-2-(phenylethynyl)pyridine (MPEP), the dynamin-dependent internalization inhibitor Dynasore, or the PKD1 inhibitor CID755673 were infused into the bilateral hippocampal CA1 areas (2 μL per side, over 5 min). The effects of these infusions and the effects of PKD1 knockdown were examined in MWM or NOD test.

**Results:**

DHPG infusion increased the latency to reach the platform in the MWM test and reduced the preference for the novel object in the NOD task. We found that the DHPG effects were dose-dependent and could be maintained for up to 2 days. Notably, these effects could be prevented by pre-infusion of the group1 mGluR antagonist MPEP, the dynamin-dependent internalization inhibitor Dynasore, the PKD1 inhibitor CID755673, or by PKD1 knockdown in the hippocampal CA1 area.

**Conclusion:**

Altogether, these findings provide direct evidence that PKD1-mediated signaling may play a critical role in the induction of learning and memory impairments by DHPG infusion into the hippocampal CA1 area.

## Introduction

Hippocampal neurons have been found to play an important role in learning and memory functions of animals and humans. Increasing amounts of data have documented that the glutamate receptor-mediated signaling in the hippocampus plays crucial roles in the regulation of learning and memory. Up- or down-regulation of NMDA type of glutamate receptors in hippocampal neurons, for example, may lead to improvements or deficits in spatial memory of rodents [1–5]. Furthermore, preventing the hypofunction of NMDARs by altering their upstream or downstream signaling pathways has been proposed as a strategy for the treatment of learning and memory dysfunction in a variety of neuropsychiatric disorders [6–8].

Highly expressed in hippocampal neurons [9], group I mGluRs (mGluR1 and mGluR5) play important roles in both long-term potentiation (LTP) and long-term depression (LTD) of AMPAR-mediated excitatory post-synaptic currents (EPSCs) [10–13]. In line with the critical involvement of the LTD and LTP of AMPAR mediated EPSCs [14–16], the down-regulation of mGluR5 may cause deficits in synaptic plasticity as well as learning and memory. For example, mGluR5 knockout leads to alterations in both LTD and LTP of AMPAR-mediated EPSCs in CA1 neurons and learning impairments [15,17,18]. Moreover, double knockout of MAPK-activated protein kinases 2 and 3 disrupts the mGluR-mediated LTD in the hippocampus of animals, and induces distinctive deficits in hippocampus-dependent spatial reversal learning [19].

The activation of Group I mGluRs may also induce LTD of NMDAR-mediated EPSCs in CA1 neurons [4], and lead the memory deficits associated with Fragile X syndrome and Alzheimer’s disease [14]. The group I mGluR depression [20,21] or NMDAR activation [22] may prevent some symptoms induced by inhibition of Fragile X Mental Retardation Protein and reduce cognitive impairment and pathogenesis in a mouse model of Alzheimer’s disease [23]. For instance, mGluR5 antagonism or genetic deletion of mGluR5 can rescue the spatial learning deficits observed [11,23]. Thus, understanding molecular mechanisms underlying the induction of learning and memory impairments remains a challenge.

Notably, recent *in vitro* investigations have shown that the activation of group I mGluRs in cultured hippocampal neurons induced by the application of (*S*)-3,5-Dihydroxyphenylglycine (DHPG, 50 μmol), a selective agonist of group I mGluRs [24], causes the regulated internalization of both AMPARs and NMDARs [11,25–28]. Furthermore, we found that such regulated internalization of surface NMDARs subsequently activates protein kinase D1 (PKD1) [25,26], which, through phosphorylating the C-terminals of the NMDAR GluN2 subunits, down-regulates the activity of remaining (non-internalized) surface NMDARs [25,26]. Notably, PKD1 blockade does not affect the inhibition of AMPAR-mediated mEPSCs induced by DHPG application but prevents the DHPG-induced down-regulation of NMDAR-mediated mEPSCs [25,26]. In light of these findings, we questioned whether a localized infusion of DHPG into the hippocampal CA1 area *in vivo* might induce any change in learning and memory behavior, as well as the underlying involvement of PKD1.

To address these questions, in this work we examined the effects of DHPG (50 μmol) infusion bilaterally into the hippocampal CA1 area *in vivo* by using the Morris water maze (MWM) and the novel object discrimination (NOD) tests. We found that the intra-CA1 infusion of DHPG caused deficits in spatial memory and formation of novel object memory, and that these effects were prevented by pre-treatment with the group I mGluR antagonist 6-Methyl-2-(phenylethynyl)pyridine (MPEP) [25,29–31], the dynamin-dependent internalization inhibitor Dynasore [32–34], the PKD1 inhibitor CID755673 [35–37], or by PKD1 knockdown.

## Materials and methods

This study was performed following the guidelines of the National Institutes of Health (NIH) and all experimental protocols were approved by the Animal Care and Use Committees at Florida State University or at Nanjing Medical University.

A total of 300 adult male Sprague Dawley rats (250–280 g) were used for behavioral tests. The rats were pair-housed with food and water available *ad libitum* except during testing, and maintained under a 12 h light/dark cycle (lights off at 7:00 PM). All behavioral experiments were conducted during the first 5 hours of the light phase of the light/dark cycle. All chemicals used in this work were obtained from Sigma (St. Louis, MO or Shanghai, China) except for those as indicated.

### Cannula implantation and chemical infusion in the CA1 area

**Fig 1:**
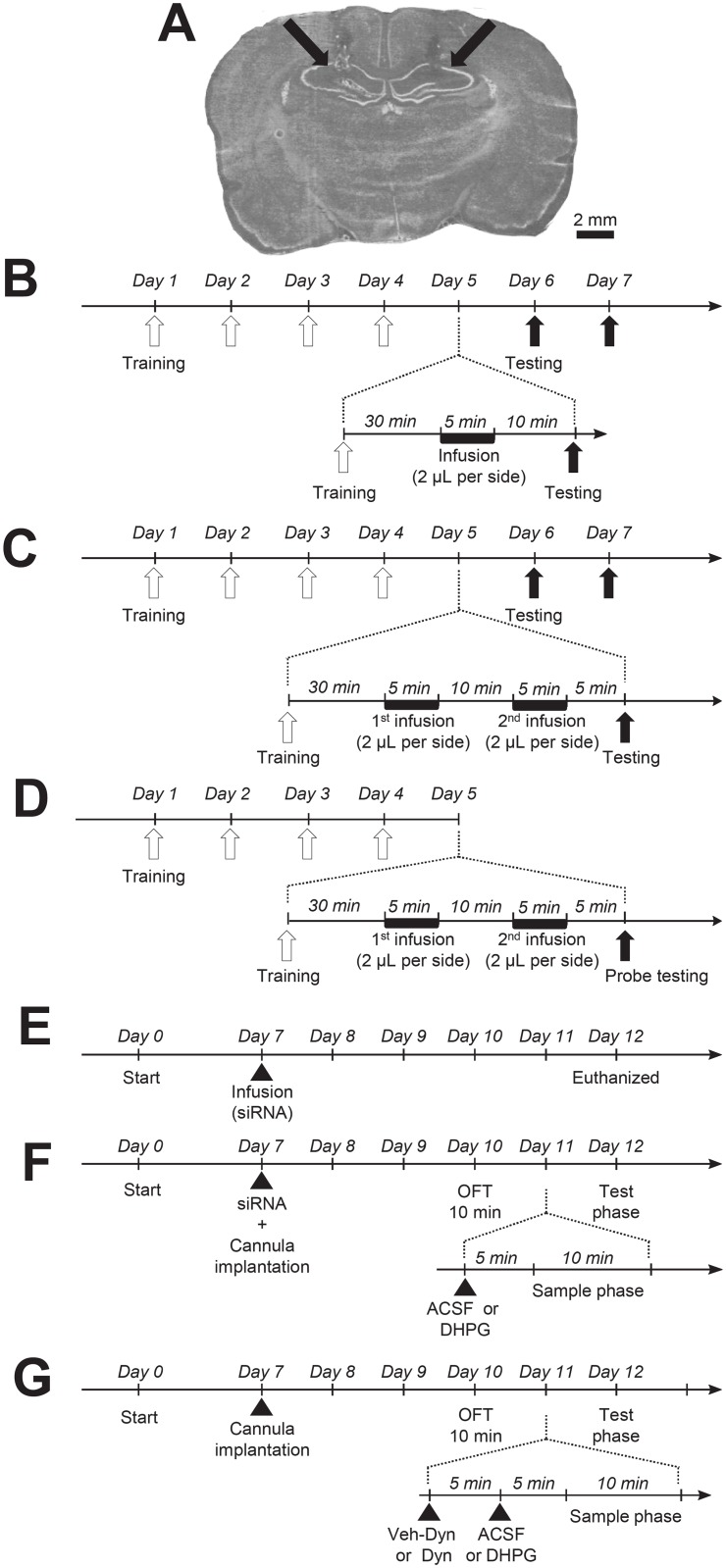
Schematics of experimental procedures. Implanted cannulas aimed at the CA1 area of the hippocampus was verified (see arrowheads) on brain section as shown in **A**. Timelines of the infusion performance in MWM test are shown in **B**, **C** and **D**. Following the recovery for 3 days from the surgery, rats were trained for MWM performance test for 5 consecutive days (see **B**, **C** and **D**). Open arrowheads in **B**, **C** and **D** indicate MWM training before the infusion, and closed arrowheads indicate testing after the infusion. Timelines of the infusion performance in NOD test are shown **E, F** and **G**. Arrowheads in **E, F** and **G** show the infusion of chemicals as indicated. siRNA: PKD1 siRNA or control siRNA; Euthanized: the animals tested were sacrificed for sampling the tissues of the hippocampal CA1 area; OFT: Open-field test; Veh-Dyn: vehicle of Dynasore; Dyn: Dynasore.

Following one week of habituation to the vivarium, rats were bilaterally implanted with cannulas aimed at the CA1 area of the hippocampus (CA1 coordinates in mm from Bregma: AP -3.14; lateral +/-2; DV -3.0). Briefly, rats were deeply anesthetized by exposure to isoflurane, placed in a stereotaxic apparatus (Kopf Instruments, Tujunga, CA) and stainless steel guide cannulas (Plastics One, Roanoke, VA) were lowered to target the coordinates listed above. Correct cannula placement was verified *a posteriori* during brain sectioning and processing (see Fig 1A). Then, the bilateral cannulas were fixed in place using dental cement and surgical anchor screws. At the end of the surgery, as described previously [38], stainless steel stylets were placed in the guide cannulas, protruding 1 mm below the end of the cannula to prevent obstruction and clogging of the guide cannula. Moreover, stylets were removed, cleaned, and replaced daily until the day of injections to help habituate the animals to the procedure and limit the stress induced by the manipulation. After the surgery rats were left undisturbed for 3 days. All rats were handled daily for post-operative monitoring and maintenance of the dummy injectors for animals implanted with cannulas. As a result, before the first behavioral test, all rats were repeatedly exposed to the experimenters and experimental procedures, and no abnormal phenotype could be detected in any group during the first exposure to the empty open field arena with regards to general locomotion and anxiety.

A previous study [39] reported that Intracerebroventricular (*i*.*c*.*v*.) infusion of DHPG immediately after the learning trial may facilitate the consolidation process in a passive avoidance situation. To examine if the intra-CA1 administration of DHPG may alter spatial memory, rats were randomly grouped after MWM training (see below) on the 5^th^ day and received bilateral infusions (2 μL per side, over 5 min) through implanted cannulas (see Fig 1B) under double blind conditions. To determine mechanisms underlying the effects of DHPG infusion, some animals received two consecutive infusions conducted 10 min apart (see Fig 1C and 1D). Rats in these groups received infusions of ACSF followed by ACSF, ACSF followed by DHPG (50 μmol), ACSF followed by the group I mGluR antagonist MPEP (10 μmol), or MPEP (10 μmol) followed by DHPG (50 μmol), ACSF followed by the PKD1 inhibitor CID755673 (182 nmol), or CID755673 (182 nmol) followed by DHPG (50 μmol). The MWM test was then performed 5 min after the second infusion (see Fig 1C and 1D). MPEP completely inhibits quisqualate-stimulated phosphoinositide hydrolysis with an IC_50_ value of 12 nmol and has no effect on other types of glutamate receptors including group II mGluRs, NMDARs, or AMPARs and kainate receptors until 100 μmol [29–31]. At the dose used in this work, 10 μmol, MPEP prevents NMDAR endocytosis induced by application of 50 μmol DHPG to cultured hippocampal neurons [25]. To determine the effect of PKD1 inhibition on the DHPG-induced alterations in MWM performances, the IC_50_ concentration (182 nmol) of the PKD1 inhibitor CID755673 [35–37] was used in this work. Notably, this dose is 100 times less than that affecting the activity of other kinases such as those of the PKC family [35–37].

For PKD1 knockdown, 1 nmol of PKD1 siRNA (TGCTGTTGACAGTGAGCGCATCGTTCACTGTGACCTCAAATAGTGAAGCCACAGATGTATTTGAGGTCACAGTGAACGATATGCCTACTGCCTCGGA) or control siRNA (TGCTGTTGACAGTGAGCGATCTCGCTTGGGCGAGAGTAAGTAGTGAAGCCACAGATGTACTTACTCTCGCCCAAGCGAGAGTGCCTACTGCCTCGGA) [25] dissolved 1:1 in GeneSilencer (Genlantis, San Diego, CA.) were infused (2 μL per side) immediately after cannulas were implanted (see Fig 1E–1G). For NOD test, after three days recovery from the surgery for cannula implantation, rats were habituated to the open field for 10 min (see Fig 1E–1G).

The following day, 1 μL of ACSF containing (in mmol): NaCl (124), KCl (3), NaH_2_PO_4_ (1.25), MgCl_2_ (2), CaCl_2_ (2), NaHCO_3_ (26), Dextrose (10) or 50 μmol of DHPG dissolved in 1 μL ACSF were infused over 3 min into the CA1 area through the implanted cannulas. *In vitro*, the DHPG effect on NMDAR activity detected appears almost immediately following its application and lasts for 15 min after its washout [25,26]. To determine whether DHPG infusion may also affect learning process, rats were exposed to the ten-minute sample phase of NOD task 5 min after the infusion, and re-exposed, twenty four hours later, to the choice phase (see Fig 1F). For the investigation of the involvement of endocytic processes in the induction of memory impairments by DHPG, 5 min prior to the infusion of ACSF containing or not DHPG, rats were injected with the cell-permeable inhibitor of dynamin, Dynasore [32,34] (80 μmol dissolved in 0.2% final DMSO in PBS) or DMSO (0.2% final in PBS, Vehicle of Dynasore) bilaterally into the CA1 areas (2 μL per side, over 5 min) (see Fig 1G). The dose and timing of injection of Dynasore were chosen based on the literature, reporting a fast (within 3–4 minutes) and strong inhibition of endocytosis in different cell types, including rodent hippocampal neurons [32], lasting for at least 20 min after its removal [33,34].

### Morris water maze (MWM) test

Following 3 days of recovery from surgery, rats were trained for 5 consecutive days (see Fig 1B and 1C) to learn the location of a hidden platform under the MWM paradigm conducted in a circular tank (150 cm in diameter, 50 cm in height) filled with colored opaque water kept at temperatures of 22–23 °C. Notably, a separate group of non-operated rats was trained and tested under identical conditions to control for eventual interference of the cannula implantation on swimming and learning performances. The pool was divided into four quadrants. The first day before training trials, rats were put into the pool and allowed to freely swim for 2 min to get familiar with the environment. For training trials, a platform (10 cm in diameter, 28 cm in height) with non-reflective interior surfaces was placed in the center of the third quadrant (Q3, the goal quadrant) and 2 cm below the water level. Rats were released respectively into water from the tank edge of 4 quadrants. If rats could not find the platform within 90 sec, they were guided to or placed on the platform for 10 sec. A trial was defined as “successfully completed” when the rat reached and then stayed on the platform for more than 10 sec. The animals were then removed from the maze for 15 min before starting the next trial. Every day, rats were trained for 4 trials. Each trial at day 5 before CA1 infusion took 16.3 ± 2.0 sec on average (*n* = 20). Thirty minutes following the 4^th^ trial on the 5^th^ day rats received the chemical infusions bilaterally into the CA1 area through implanted cannulas (see Fig 1B). Ten minutes after the infusion, the same MWM trials were conducted and repeated for two subsequent days (see Fig 1B). The probe test session was conducted after the CA1 infusion at day 5 (see Fig 1D). In this session, the platform was removed and rats were allowed to freely swim for 90 sec. Rat behavior during training and testing was video-recorded for further analyses using the software MWM analysis system (Zhenghua Biological Instrument Equipment Co., Suixi, Anhui). For evaluation of memory functions, the average swimming speed, the latency to reach the platform, the percentage of swimming distance or time within the goal quadrant relative to the total distance or time, as well as the number of platform location crossings in the probe test session, were measured.

### Novel object discrimination (NOD) test

The NOD test was performed as described previously [40–43]. In brief, the NOD testing apparatus was a regular open field with black Plexiglas floor and walls (90 cm x 90 cm), located in a room illuminated with dim white light (30 LUX). The objects used for discrimination were a square glass jar filled with sand (object A) and a steel cylinder filled with sand (object B), which were of different shapes and texture. The objects held no ethological significance for the rats and had never been seen by the rats prior to testing. A preliminary experiment was carried out to ensure that rats showed no preference for one object over the other (data not shown).

On the first day of testing, all rats were habituated to the empty open field apparatus for 10 min. On the next day of testing, rats were exposed to a sample phase, which was followed by a choice phase twenty four hours later (see Fig 1E and 1F). During the sample phase, rats were placed in the open field and allowed to investigate two identical copies of the same “sample-object” (*e*.*g*., glass jar) for 10 min. Objects were placed diagonally in opposite sides of the open field, near the corners of the apparatus. After the 10 min sample phase, rats were returned to their home cages (see Fig 1E and 1F). During the choice phase, one of the “sample” objects was replaced by a “novel object” (*e*.*g*., steel cylinder), and the positions of all objects were counterbalanced between rats and between sessions in order to avoid positional bias. Both the objects and the open field apparatus were thoroughly wiped clean between trials with 70% ethanol in order to get rid of olfactory cues.

Rat behaviors during all sessions were video-recorded and quantified using EthoVision XT 8 (Noldus Information Technology, Leesburg, VA). The total distance moved, speed, time spent in the center of the arena, as well as time spent in its corners, were thus measured during the first (open-field) session, whereas the time spent exploring objects were quantified during both the sample and the test phase. “Exploration of an object” was defined as directing the nose to the object at a distance of less than 2 cm and/or touching the object with its nose. Behaviors such as climbing or using the objects for leverage, as well as any other type of bodily contact, were not considered as exploration. Data were expressed as the percentage of time spent exploring the novel object relative to the total exploration time, defined as (*T*B / (*T*A + *T*B) x 100). A value of 50% thus corresponds to chance level and a significantly higher percentage reflects good recognition memory.

### Rotarod test

To determine if the intra-CA1 infusion of drugs used in our study might alter motor functions, the rotarod performance test was conducted as described previously [44]. In brief, after a 3 day recovery period from the cannula surgery, 8 rats were trained on a rotating rod apparatus (DigBehv-RRTR, Jiliang Biomart, Shanghai) twice a day for 3 consecutive days. The rotation speed was 5 rpm at the beginning, and then increased to 15 rpm within 120 sec before being maintained for 180 sec. Before DHPG infusion on the fourth day of the test, the time spent on the rotating rod (latency to fall) was measured 3 times with an intertrial interval of 30 min, and the mean value of the 3 measurements was calculated for each rat. Fifteen minutes after DHPG infusion, rats were subjected to 3 additional trials, and the averaged latency to fall was calculated for each rat.

### Western blot

Rats were euthanized immediately following the test phase by rapid decapitation, their brain dissected out and snap-frozen in 2-methyl butane chilled to -30 °C before being stored at -80 °C until further processing. Total protein lysates were extracted from the CA1 area of the hippocampus at the injection site using Tri-Reagent (Molecular Research Center, Inc. Cincinnati, OH) according to the manufacturer’s instructions. Following spectrophotometric quantification of total protein concentrations (Nanodrop, Thermo Scientific, Waltham, MA), 10 μg of the protein were loaded on a 12% sodium dodecyl sulfate polyacrylamide gel and transferred onto a nitrocellulose membrane before incubation with primary antibodies directed against PKD1 [45] (diluted 1:1000, Cell Signaling, Beverly, MA) or GAPDH (1:5000, Cell Signaling). After incubation with corresponding secondary antibodies and signal detection, the signal for PKD1 was quantified using ImageJ (NIH), and normalized to the GAPDH signal of the same sample on the same membrane. For easier comparison between the control siRNA and PKD1 siRNA groups, all normalized ratios were transformed to 100% of controls—set as the control siRNA group—and plotted as such. All statistical tests were performed on the untransformed normalized ratios in order to avoid any interference of the transformation step.

### Statistic analysis

**Fig 2:**
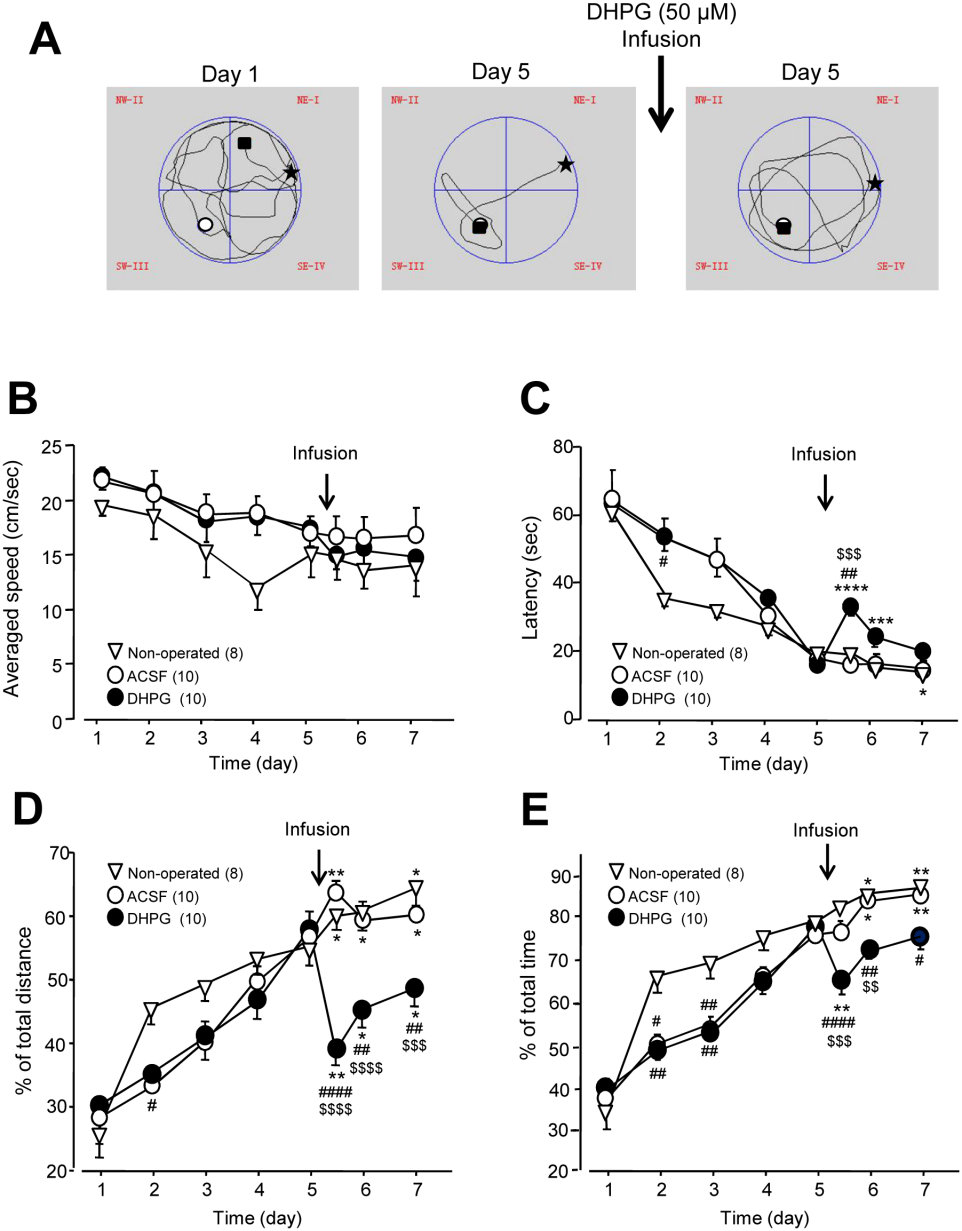
Impairments of spatial memory induced by bilateral infusions of DHPG (50 μmol) into the CA1 area. **A** shows swimming track plots of a rat in the MWM performance test in a training session at day 1 (left) and day 5 before (middle) and after (right) the infusion of DHPG (50 μmol) into the bilateral CA1 areas, respectively. Arrow indicates the intra-CA1 infusion of DHPG. Stars indicate where the rat was put into the pool; white circles indicate the position of a platform in the third quadrant (Q3, the goal quadrant) and 2 cm below the water level. Black squares indicate the final locations of the rat. The summary data (mean ± SEM) of the latency from when rats were released into the pool to reaching the platform, the averaged swimming speed, the percentages of swimming distance and time within the goal quadrant relative to the total swimming distance and time are shown in **B**, **C**, **D** and **E**, respectively. Open triangles indicate the performance of non-operated rats. Arrows indicate the infusion of ACSF (open circles) or DHPG (filled circles) into the bilateral CA1 areas. *: *p* < 0.05, **: *p* < 0.01. ***: *p* < 0.001, ****: *p* < 0.0001 (paired *t*-test) in comparisons with that before the infusion at day 5 in the ACSF or DHPG group, or with that of the first performance of MWM at day 5 in non-operated rats; #: *p* < 0.05, ##: *p* < 0.01, ####: *p* < 0.0001 (Bonferroni post hoc test in repeated measures two-way ANOVA) in comparison with those of the non-operated group; $: *p* < 0.05, $ $: *p* < 0.01, $ $ $: *p* < 0.001, $ $ $ $: *p* < 0.0001 (Bonferroni post hoc test in repeated measures two-way ANOVA) in comparison with those of the ACSF group. See S1 Table for statistical details; Values in the brackets indicate the number of rats tested.

**Fig 3:**
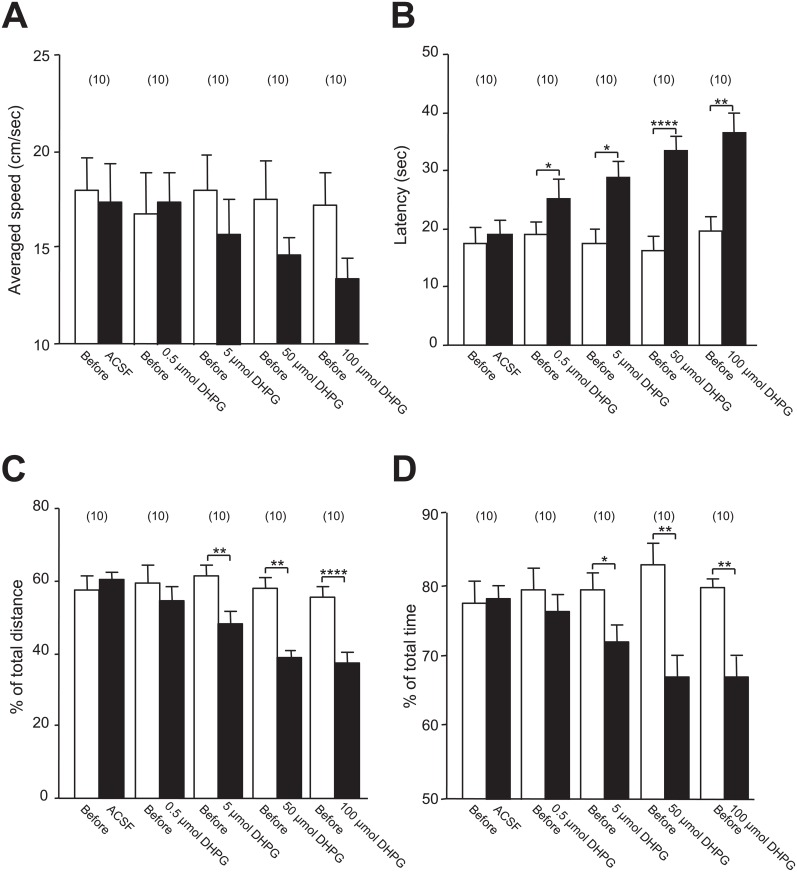
Dose-dependent changes in spatial memory induced by DHPG infusion. Bar graphs in **A, B, C** and **D** respectively show the summary data (mean ± SEM) of the averaged swimming speed, latency and the percentages of swimming distances and time within the goal quadrant relative to the total swimming distance and time before (open bar) and after the infusion of ACSF or 0.5, 5, 50 or 100 μmol DHPG as indicated (Closed bar) in the MWM performance test. *: *p* < 0.05, **: *p* < 0.01, ****: *p* < 0.0001 (paired *t*-test) in comparison with that before the infusion. See S2 Table for statistical details; Values in the brackets indicate the number of rats tested.

**Fig 4:**
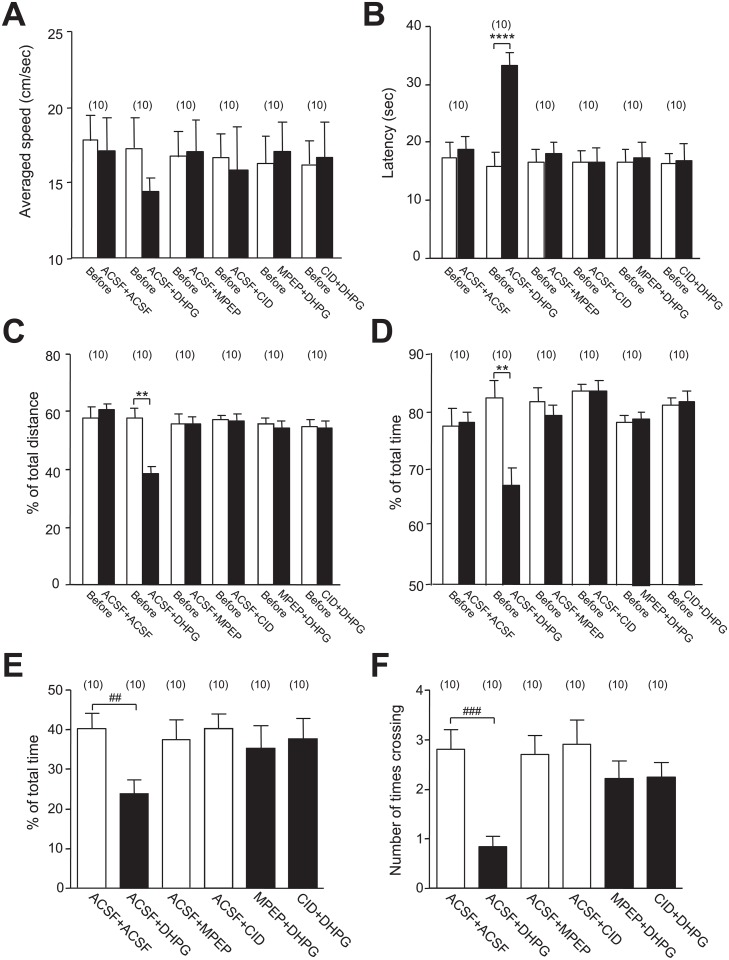
Pre-infusion of MPEP or CID755673 prevented impairments of spatial memory induced by DHPG infusion. Bar graphs in **A**, **B**, **C** and **D** show the summary data (mean ± SEM) of the averaged swimming speed, latency, and percentage values of the swim distance and time in the goal quadrant versus the total swimming distance and time of rats in the MWM test. Bar graphs in **E** and **F** show the summary data (mean ± SEM) of the percentage values of the swimming time spent in the goal quadrant, and the number of times crossing the location of the platform in the probe test. Rats received two infusions of chemicals through implanted cannulas in the bilateral CA1 areas as indicated. The second infusion was conducted 10 min after the completion of the first one. The MWM test was performed 5 min after the second infusion. ACSF+ACSF: receiving two infusions of ACSF; ACSF+DHPG: receiving infusions of ACSF followed by DHPG (50 μmol); ACSF+CID: receiving infusions of ACSF followed by CID755673 (182 nmol); MPEP+DHPG: receiving infusions of MPEP (10 μmol) followed by DHPG (50 μmol); CID+DHPG: receiving infusions of CID755673 (182 nmol) followed by DHPG (50 μmol). **: *p* < 0.01, ****: *p* < 0.0001 (paired *t*-test) in comparison with that before the pre-infusion; #: *p* < 0.05, ###: *p* < 0.0001 (unpaired *t*-test) in comparison with rats received ACSF+ACSF. See S3 Table for statistical details; Values in brackets indicate number of animals tested.

**Fig 5:**
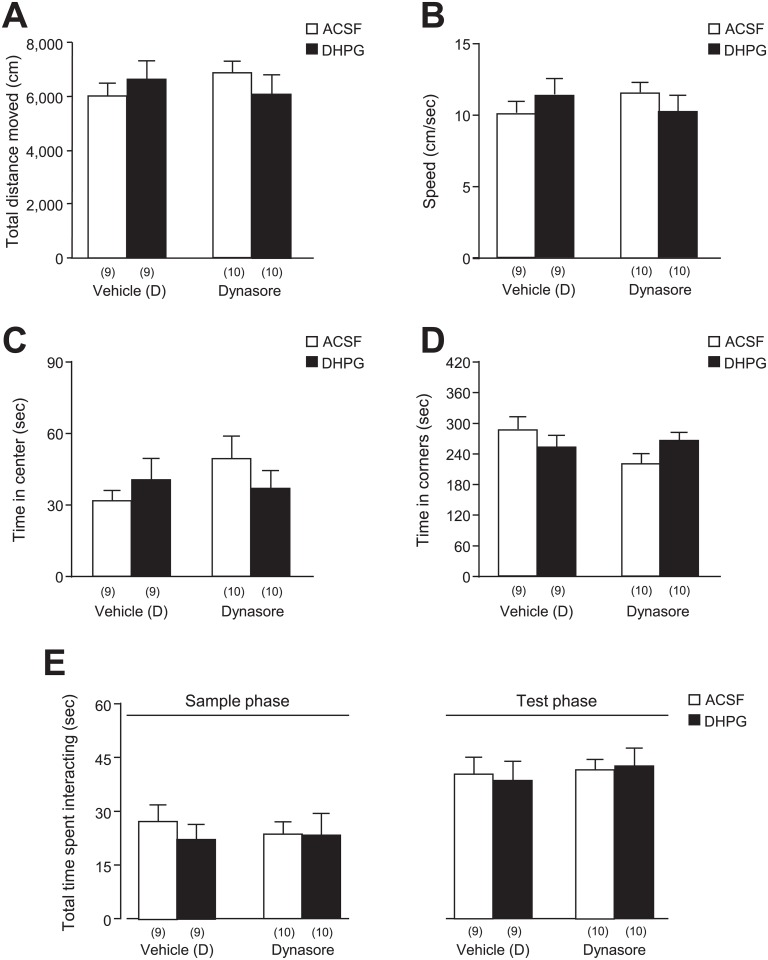
General locomotion, anxiety, and investigative behaviors of rats tested for the DHPG effects. The general locomotor activity and anxiety levels of rats undergoing the NOD task was first tested in the empty arena during an open-field session. The total distance moved (**A**), speed (**B**), time in center (**C**), as well as the time spent in all four corners of the arena (**D**) were thus quantified in rats that would be infused with ACSF or DHPG, after pre-infusion of Dynasore or its vehicle [Vehicle (D)]. Furthermore, the total time spent interacting with either objects during the sample phase and the test phase of the NOD task is depicted in **E**. See S4 Table for statistical details; Values in the brackets indicate the number of rats tested.

**Fig 6:**
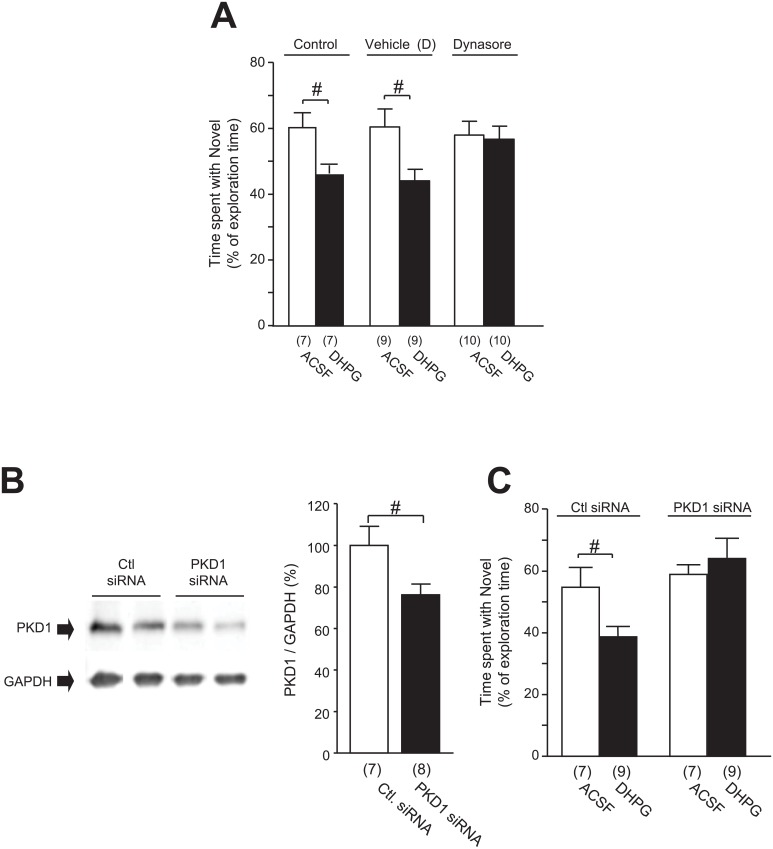
Effects of bilateral infusion of DHPG (50 μmol/2 μL) into the CA1 area on the NOD performance. The preference for the novel object is represented by the time spent interacting with the novel object as a percentage of the total time spent interacting with objects in **A** for rats following the infusion of ACSF or DHPG into the CA1 area without (Control, left two bars in **A**) or with co-application of vehicle of Dynasore (middle two bars in **A**) or Dynasore (80 μmol, right two bars in **A**). Blots in **B** show examples of Western blot analysis of PKD1 (upper blots) and GAPDH proteins (lower blots) in the CA1 area in rats which received the infusion of control siRNA (Ctl. siRNA) or PKD1 siRNA in this area. Summary data of the ratios of band intensities of PKD1 versus GAPDH proteins (= 100%) are shown in the bar graphs in **B**. Bar graphs in **C** show the preference for the novel object—represented by the time spent interacting with the novel object as a percentage of the total time spent interacting with objects—of rats following intra-CA1 infusion of ACSF (open bars) or DHPG (filled bars) pre-administrated with PKD1 siRNA or control siRNA. #: *p* < 0.05 (unpaired t-test); See S5 Table for statistical details; Values in brackets indicate the number of animals tested.

In order to determine which type of statistic tests should be used, we performed multiple tests for assessing data normality or variance. ANOVA as well as unpaired or paired *t*-tests were used in this work. All data are expressed as mean ± SEM and a *p*-value < 0.05 was considered statistically significant. Detailed information of statistical tests conducted for data shown in Figs 2–6 is presented in S1–S5 Tables.

## Results

We examined the effects of bilateral infusions of DHPG into the hippocampal CA1 area on the spatial memory of rats in the MWM task under double-blind conditions (see Fig 2). After 5 days of training, rats were randomly distributed into two groups and bilaterally received chemical infusions through implanted cannulas targeting the CA1 areas (see Fig 1B) thirty minutes after the 4^th^ trial on the 5^th^ day. Ten minutes later, the same MWM trials were repeated for two subsequent days (see Fig 1B).

During the initial training period of the MWM test—from day 1 to the first session of day 5—the swimming speed was progressively reduced over time in non-operated rats (*p* = 0.03, see Fig 2B, and S1 Table) but not in cannula-implanted rats (*p* = 0.35 before ACSF, *p* = 0.32 before DHPG; see Fig 2B, and S1 Table). However, no statistically significant difference could be found among the three groups (*p* = 0.90 for interaction; see Fig 2B and S1 Table). The latency to reach the platform was progressively reduced over time in all three groups (*p* < 0.0001 in the non-operated group, *p* < 0.0001 before ACSF group, *p* < 0.0001 before DHPG; see Fig 2C and S1 Table). Furthermore, we examined the relationship between the swimming speed and the latency to reach the platform. No statistically significant correlation was found between the latency and the swimming speed in all the groups during the training period (non-operative: pearson r = 0.127, *p* = 0.436; before ACSF: pearson r = 0.045, *p* = 0.755; before DHPG: pearson r = 0.121, *p* = 0.286; see S1A–S1C Fig).

The percentages of the swimming distance (Fig 2D) and time (Fig 2E) in the goal quadrant increased (the percentage of swimming distance: *p* < 0.0001 in the non-operated rat group, *p* < 0.0001 before ACSF, *p* < 0.0001 before DHPG; percentage of swimming time: *p* < 0.0058 in the non-operated rat group, *p* < 0.0001 before ACSF, *p* < 0.0001 before DHPG; see S1 Table). Although statistically significant differences were noted between the three groups at day 2 and/or day 3 of the training session (Fig 2C–2E and S1 Table), on the 4^th^ and 5^th^ days no difference in the latency or percentages of the swimming distance and time could be found between the three groups of rats (Fig 2C–2E). All three groups thus displayed improvement of their performances in the MWM over repeated training sessions, denoting correct acquisition of the learning task.

Following ACSF infusions, although the averaged swimming speed remained unaffected (*p* = 0.81 at day 5, *p* = 0.85 at day 6, *p* = 0.97 at day 7; see Fig 2B and S1 Table), performances in the MWM test continued to improve when compared to day 5 before infusions, as reflected by the increase in the percentages of swimming distance and time spent in the goal quadrant during subsequent trials (the percentage of swimming distance: *p* = 0.0011 at day 5, *p* = 0.036 at day 6, *p* = 0.021 at day 7; the percentage of swimming time: *p* = 0.54 at day 5, *p* = 0.023 at day 6, *p* = 0.002 at day 7; see Fig 2D and 2E and S1 Table). Notably, these performances were similar to non-operated rats measured at corresponding times (Fig 2B–2E), indicating that the infusion method used and ACSF did not produce any effect.

Following infusion of the group I mGluRs agonist DHPG, no significant change in the swimming speed was observed at the same day when compared to that before DHPG infusion (*p* = 0.35, see Fig 2B and S1 Table), or to those in non-operated or ACSF-infused animals (*p* = 0.99 for interaction; see Fig 2B and S1 Table). However, the latency to reach the platform was significantly increased following DHPG infusion (*p* < 0.0001, see Fig 2C and S1 Table), and longer than that of non-operated (*p* < 0.01, see Fig 2C and S1 Table) or ACSF-infused rats (*p* < 0.0001, see Fig 2C and S1 Table). Moreover, the percentages of swimming distance and time spent in the goal quadrant are both reduced following DHPG infusion (the percentage of distance: *p* = 0.0025; the percentage of time: *p* = 0.001; see Fig 2D and 2E and S1 Table), and lower than that of non-operated (the percentage of distance: *p* < 0.0001; the percentage of time: *p* < 0.001; see Fig 2D and 2E and S1 Table) or ACSF-infused rats (the percentage of distance: *p* < 0.0001; the percentage of time: *p* < 0.001; see Fig 2D and 2E and S1 Table). Notably, such DHPG effects were maintained for the subsequent days (one for the increased latency to reach the platform, two for the percentage of distance and time spent in the goal quadrant, Fig 2C–2E). Altogether, these observations revealed impairments in MWM performances following DHPG infusion into the hippocampal CA1 area, suggesting a deficit in the retrieval of spatial memory.

In order to further analyze the effects of intra-CA1 infusion of DHPG on the spatial memory, we then examined the dose-dependent effects of DHPG on MWM performances after bilateral infusions at 0.5, 5, 50, or 100 μmol under the same experimental design as described above (Fig 1B). Despite a trend for reduced swimming speed with increasing DHPG concentrations, no statistically significant change was found when compared with pre-DHPG speed, even at the highest concentration 100 μmol (*p* = 0.16; see Fig 3A and S2 Table). Moreover, no statistically significant negative correlation between the latency and the swimming speed was found in any group except the group of rats after ACSF infusion (see S1 Fig). When compared with naïve or ACSF-infused rats, this association was reduced in DHPG-infused rats (see S1 Fig). These findings thus suggest that after DHPG infusion, the increase in latency to reach the platform is less likely to be due to the reduction in the swimming speed.

Interestingly, while the 5, 50, and 100 μmol doses altered performances in the MWM—as reflected by increased latencies to reach the platform (Fig 3B) and reduced percentages of distance (Fig 3C) and time (Fig 3D) spent in the goal quadrant—, the dose of 0.5 μmol failed to reduce the percentage of distance (*p* = 0.45; see Fig 3C and S2 Table) and time spent in the goal quadrant (*p* = 0.39; see Fig 3D and S2 Table). Thus, we conclude that the bilateral infusions of DHPG into the CA1 area may induce dose-dependent deficits in spatial memory.

Recent investigations have shown that the activation of group I mGluRs by DHPG application causes the regulated internalization of NMDARs [11,12,25–28]. We found that the regulated NMDAR internalization induced by DHPG application in cultured hippocampal neurons may activate PKD1, which subsequently phosphorylates the C-terminals of the NMDAR GluN2 subunits and down-regulates the NMDAR-, but not AMPAR-, mediated synaptic responses [25,26]. We therefore investigated whether the DHPG-induced impairments in spatial memory may be affected by group1 mGluR antagonism or PKD1 inhibition. Effects of bilateral pre-infusions of ACSF, the group I mGluRs antagonist MPEP (10 μmol), or the PKD1 inhibitor CID755673 (182 nmol) into the CA1 area were examined (Fig 4). Ten minutes after the completion of the pre-infusions, the second infusions were conducted in the same CA1 areas, followed by MWM sessions performed 5 min later (see Fig 1C and Methods). Similar to the findings reported above (Figs 2 and 3), DHPG-treated animals pre-infused with ACSF (ACSF+DHPG group) exhibited increased latencies to reach the platform (*p* < 0.0001; see Fig 4B and S3 Table) and reduced percentages of swimming distance (*p* = 0.0016; see Fig 4C and S3 Table) and time (*p* = 0.0022; see Fig 4D and S3 Table) in the goal quadrant when compared with those before the pre-infusions. By contrast, no such effects were observed after the treatment with ACSF (ACSF+ACSF), MPEP (ACSF+MPEP), or CID755673 (ACSF+CID) following the ACSF pre-infusion (see Fig 4 and S3 Table). Notably, DHPG effects on these behavioral outcomes were blocked by MPEP or CID755673 pre-treatment (see Fig 4 and S3 Table), suggesting that the spatial memory impairments induced by DHPG may be mediated by group I mGluRs and PKD1.

To confirm this finding, the effects of pre-infusion of ACSF, MPEP or CID755673 into the CA1 area were examined with probe tests (Fig 1D). DHPG-infused animals (ACSF+DHPG) displayed reduced percentage of time spent in the target quadrant (*p* = 0.005; see Fig 4E and S3 Table) and number of platform location crossing (*p* = 0.0004; see Fig 4F and S3 Table) when compared with ACSF-infused rats (ACSF+ACSF). Notably, in accordance with our observations reported above, this effect was also prevented by pre-infusion of MPEP or CID755673 (see Fig 4E and 4F and S3 Table), further indicating that the intra-CA1 infusion of DHPG may induce memory impairment through group I mGluRs and PKD1.

In an effort to further analyze DHPG effects on learning and memory processes under a paradigm with a lower stress component than the MWM procedure, we then investigated the formation of a novel object recognition memory. Following bilateral implantation of guide cannulas into the hippocampal CA1 area, rats were thus subjected to a three-session novel object discrimination (NOD) test (Fig 1F and 1G). During the first exposure to the empty open field arena—prior to any drug infusion—, no abnormal phenotype was detected in any group with regards to general locomotion and anxiety (Fig 5)—as reflected by the total distance moved (*p* = 0.999 for DHPG, *p* = 0.882 for Dynasore, *p* = 0.214 for interaction; see Fig 5A and S4 Table), speed (*p* = 0.999 for DHPG, *p* = 0.890 for Dynasore, *p* = 0.212 for interaction; see Fig 5B and S4 Table), time spent in the center of the arena (*p* = 0.784 for DHPG, *p* = 0.320 for Dynasore, *p* = 0.158 for interaction; see Fig 5C and S4 Table), and time spent in corners (*p* = 0.833 for DHPG, *p* = 0.202 for Dynasore, *p* = 0.046 for interaction; see Fig 5D and S4 Table)—indicating that the cannula implantation was not altering the rats’ ability to explore the arena. In line with these observations, bilateral DHPG infusions into the CA1 area did not affect the latency to fall in the Rotarod test (before DHPG: 143.9 ± 30 sec; after DHPG: 167.2 ± 32 sec; *n* = 8, *t*_7_ = 1.0, *p* = 0.340, paired *t*-test), denoting similar motor coordination before and after DHPG-infusion. Notably, all rats exhibited good exploration of objects in both the sample and test phase of the test (sample phase: *p* = 0.556 for DHPG, *p* = 0.798 for Dynasore, *p* = 0.588 for interaction; test phase: *p* = 0.921 for DHPG, *p* = 0.510 for Dynasore, *p* = 0.739 for interaction; see Fig 5E and S4 Table), indicating that none of the drug treatments affected the animals’ exploration and investigation of the objects, thereby ruling out interference with learning and memory performances.

During the test phase of the NOD task, however, only ACSF-, but not DHPG—infused rats (50 μmol/2 μL) exhibited a preference for the novel object (in control group *p* = 0.037 for ACSF, *p* = 0.965 for DHPG; see Fig 6A and S5 Table). Moreover, DHPG-infused rats spent significantly less time exploring the novel object than their ACSF-infused counterparts (59.1 ± 4.2% for ACSF and 45.6 ± 2.0% for DHPG in control group; *p* = 0.014; see Fig 6A and S5 Table). The extended time spent with the familiar object in DHPG-treated rats indicated a lack of recognition of the novel object, demonstrating that the DHPG treatment caused impairments of object recognition memory formation.

Furthermore, such DHPG effects were prevented by pre-treatment with Dynasore (80 μmol), which blocks dynamin-dependent internalization processes [32,34]. While DHPG treatment significantly reduced the percentage of time spent with the novel object in animals receiving the vehicle for Dynasore [Fig 6A Vehicle (D); ACSF: 60.5 ± 5.4%, DHPG: 44.2 ± 3.5%], DHPG infusion produced no effect in rats pre-infused with Dynasore (Fig 6A Dynasore; ACSF: 58.1 ± 4.3%, DHPG: 56.8 ± 4.0% in DHPG group). Similarly, rats pre-treated with the vehicle for Dynasore and infused with ACSF presented with a preference for the novel object (*p* = 0.043; see Fig 6A and S5 Table), that was lost in DHPG-treated rats (*p* = 0.935; see Fig 6A and S5 Table). In Dynasore-treated rats, however, the preference for the novel object remained high in both ACSF- and DHPG-infused animals (*p* = 0.044 for ACSF, *p* = 0.061 for DHPG; see Fig 6A and S5 Table). These findings suggest that endocytic processes are involved in the impairments of the memory formation induced by DHPG infusion.

We then examined the role of PKD1 in the hippocampal CA1 area by testing whether PKD1 knockdown could interfere with the DHPG effect on the formation of object recognition memory. Compared to infusion of control siRNA, PKD1 siRNA infusion selectively reduced the expression of PKD1 (*p* = 0.036, see Fig 6B and S5 Table). Interestingly, while PKD1 siRNA in the CA1 area did not affect the preference for the novel object following ACSF injection, it blocked the DHPG-induced impairments (ACSF: 57.2 ± 3.0%, DHPG: 63.7 ± 5.9%, *p* = 0.543; see Fig 6C and S5 Table), resulting in both groups exhibiting a preference for the novel object (*p* = 0.014 for ACSF: *t*_*6*_ = 3.05, *p* = 0.024 for DHPG; see Fig 6C and S5 Table). In contrast, although neither group presented with a significant preference for the novel object, the DHPG-induced reduction in time spent with the novel object was preserved in animals pre-treated with control siRNA (ACSF: 54.6 ± 6.5%; DHPG: 39.5 ± 3.1%; *p* = 0.040; see Fig 6C and S5 Table). This data thus indicates that PKD1 may also be a necessary factor in the DHPG-induced deficits in object memory formation.

## Discussion

We previously showed that stimulating group I mGluRs in cultured hippocampal neurons by bath application of DHPG (50 μmol) or stimulating NMDAR receptors by application of NMDA (1 mmol) and glycine (100 μmol) not only reduces the surface expression of NMDARs but also inhibits the activity of remaining (non-internalized) surface NMDARs [25,26]. Notably, this regulation is mediated by the activation of PKD1, which in turns phosphorylates GluN2 subunits of remaining surface NMDARs and thereby down-regulates their activity [25,26]. Furthermore, the inhibition of remaining surface NMDARs underlies the down-regulation of NMDAR-mediated synaptic responses following NMDAR endocytosis induced by DHPG or NMDA/glycine [25,26]. The inhibition of dynamin-dependent internalization by application of the peptide Myr-4–QVPSRPNRAP (50 μmol) [46–48] not only prevents DHPG- or NMDA/glycine-induced reduction of the number of NMDARs expressed on neuronal surface but also prevents both the activation of PKD1 and the down-regulation of remaining surface NMDARs [25,26]. Moreover, inhibiting PKD1 by the application of a protein kinase inhibitor such as Staurosporine, or by the knockdown of PKD1 through infection of PKD1 shRNA does not affect the regulated NMDAR endocytosis but prevent NMDAR endocytosis-induced phosphorylation and inhibition of remaining NMDARs [25,26]. Our present data show that the intra-CA1 infusion of DHPG significantly increased the latency to reach the platform, reduced the percentages of swimming distance and time in the goal quadrant, and the number of times crossing the location of the platform in MWM tests when compared with pre-DHPG trials and/or ACSF infusion, indicating impairments in spatial memory. Pre-infusion of the group I mGluRs antagonist MPEP, the inhibitor of dynamin-dependent internalization, Dynasore, PKD1 inhibitor CID755673 or the knockdown of PKD1 could all prevent the effects of DHPG on spatial memory or novel object memory formation. These findings are in line with our previous *in vitro* studies and provide the first line of direct *in vivo* evidence that infusion of DHPG into the CA1 area may induce deficits in spatial memory and the formation of object recognition memory through similar mechanisms. Furthermore, as the effects induced by the intra-CA1 infusion of DHPG could be observed for up to two days (see Fig 2C–2E), it is possible that such pre-training infusion of DHPG might affect both the acquisition and consolidation of memory.

Although statistically significant differences were noted at days 2 and 3 of the training session when compared to non-operated rats, no such differences could be found between the three groups of rats in the latency and the percentages of the swimming distance and time on the later stage—the 4^th^ and 5^th^ days (Fig 2C–2E). Thus, all three groups displayed improvement of their performances in the MWM over repeated training sessions, denoting correct acquisition of the learning task. Notably, performances of non-operated versus ACSF-infused rats were similar, indicating that the intra-CA1 infusion method used and ACSF did not affect spatial memory.

Similarly, the DHPG dose and injection method used in the present study did not affect the rats’ locomotor activity in an open-field, latency to fall in the Rotarod test, swimming speed in the MWM, or time spent in the center or investigating objects in the open-field arena. Thus, alterations in learning and memory indicators following DHPG infusion are not likely to result from non-specific effects on general locomotion, motor coordination, as well as exploratory and investigative behaviors.

Previous studies have shown that CA1 pyramidal cells exhibit place-related firing in the water maze [49] and that the excitability of CA1 neurons can be enhanced by water maze learning [50]. The spatial and temporal aspects of memory may be integrated within CA1 neuronal networks [1,51,52]. Following a discrete lesion in the CA1 area, recent and remote memory in the water maze task are found to be similarly impaired in context fear conditioning or in trace fear conditioning [53]. Moreover, pharmacological blockade [1,54–56] or genetic deletion [1,56–60] of NMDARs in hippocampal CA1 neurons have been found to interfere with acquisition, memory encoding, consolidation, and retrieval processes. However, conflicting results regarding the involvement of NMDARs in hippocampal CA1 neurons were also reported. For example, chronic infusion of the NMDAR antagonist AP5 for 7 days following water maze training does not to affect memory retention [61], whereas mice lacking the GluN1 subunit of NMDARs in the dentate gyrus and CA1 regions of hippocampus performed the water maze task as well as controls [62,63]. Thus, understanding the role of NMDA receptors of hippocampal CA1 neurons in spatial learning and memory remains a challenge.

A common concern about the MWM paradigm is the potential for interference of stress with the behavioral readout [56]. We thus chose to further investigate the DHPG effects on the formation of novel object recognition memory in the NOD paradigm. Despite the consensus that hippocampal activity is not critical for object recognition memory [64–66], several studies do report an involvement of hippocampal functions in certain aspects of the novel object memory or under certain conditions—thereby suggesting that hippocampal activity can be a modulator [67–73]. In particular, pharmacological blockade of mGluR1 receptors or *GluN1* gene knockout both result in impairments in novel object recognition memory [72,73], which is of particular interest in the context of our working model linking group I mGluRs activation to NMDAR function. As a result, we chose to further test our hypothesis in the NOD task by following an experimental design allowing for the investigation of NMDAR-mediated alterations in hippocampal function [43].

A previous study has reported that *i*.*c*.*v*. infusion of DHPG dissolved in 0.9% NaCl at doses of 25, 50 and 100 nmol immediately after the learning trial facilitated the consolidation process in a passive avoidance situation [39]. If the drug was given before the learning trial or before the retention testing, however, it did not significantly affect the acquisition or retrieval processes. Moreover, *i*.*c*.*v*. infusion of DHPG did not affect object recognition memory evaluated in the novel object discrimination (NOD) test [39].

It is known that drug doses and delivery ways can be important factors affecting the drug effects. Our present data show that DHPG at 50 μmol, which causes the regulated internalization of AMPARs and NMDARs *in vitro* [11,25–28], infused specifically into the CA1 area lead to deficits in the formation of object memory in the NOD task. We found that following infusion of 0.5 μmol DHPG into the CA1 area, only the latency for reaching the platform in the MWM test showed a significant increase when compared pre-infusion performances in the MWM test (Fig 3). When the concentration of DHPG was increased to 5 μmol or higher, however, in addition to the latency, the percentages of swimming distance and time spent in the goal quadrant were also affected (Fig 3). As a result, our data indicate that the DHPG-induced memory impairments are dose-dependent.

Group I mGluRs (mGluR1 and mGluR5) have been found to be involved in multiple actions on neuronal functions through G-protein-linked modifications of enzymes and ion channels. Activation of group I mGluRs may activate protein kinase C and mobilize calcium from intracellular stores via the up-regulation of the metabolism of phosphatidylinositol phosphate to diacyloglycerol and inositol triphosphate [74,75]. Group I mGluRs may undergo internalization following their agonist stimulation, and thereby causing their desensitization [76–78]. The scaffolding protein spinophilin and protein phosphatases PP2A and PP2B have been found to play important roles in the regulation of group I mGluR trafficking [76–78]. Group I mGluRs may act pre-synaptically to modify transmitter release or post-synaptically to regulate NMDARs and AMPARs [11–13,16,79–81], and thereby are critically involved in learning and memory functions [14–16]. It has been found that novel object recognition may facilitate hippocampal LTD recorded *in vivo* [82,83] and that mGluR5 and NMDARs are required for this facilitation [84]. mGluR5 knockout leads to deficits in both LTD and LTP of AMPAR-EPSCs in CA1 neurons and impairs learning [15,17,18]. Different from those found in the hippocampal CA1 area [25–28], the stimulation of group I mGluRs with DHPG in striatal neurons of adult rats may increase expression of GluN1 and GluN2B subunit of NMDARs on the neuronal surface [85]. The status of group I mGluR activation induced by endogenously-released ligands, such as glutamate, can be different from that induced by infusion of exogenous agonists, such as DHPG. Thus, the intra-CA1 DHPG infusion-induced effect may not be same as that produced by the activation of mGluR1s and/or mGluR5s observed under different experimental conditions. Therefore, the intra-CA1 DHPG infusion-induced effect might imply a new mechanism leading to learning and memory impairments. In our present study, no alteration in MWM performance was observed following the intra-CA1 infusion of MPEP (10 μmol in 2 μL per side), which may seem in contradiction with the existing literature [29,86–88]. Indeed, systemic injections (*i*.*p*.) of MPEP at 6 mg/kg or more may impair visuo-spatial discrimination in intact mice [86], whereas its *i*.*c*.*v*. infusion at 1.8 μg for several days alters memory performance in the 8-arm radial maze [29,87,88]. Interestingly, MPEP administration in a novel environment may reduce place cell firing and spatial information but has no effect on place field profiles in a familiar environment [89]. Altogether, these data suggest that the effects of MPEP may be highly dependent on the dose, delivery method, and experimental conditions.

Previous studies including ours have shown that application of DHPG to hippocampal neurons causes internalization of both AMPARs and NMDARs [11,25–28], and down-regulates synaptic responses mediated by either AMPARs or NMDARs [4,11,25–28]. Although the contribution of either AMPARs, or NDMARs—or both—in the *in vivo* DHPG-induced learning and memory impairments reported here remains to be elucidated, the observation that PKD1 knockdown prevented such learning and memory impairments brings novel insight for understanding the mechanism underlying DHPG-induced impairments of learning and memory. Indeed, the DHPG-induced inhibition of AMPAR-mediated mEPSCs is not affected by PKD1 knockdown, which, however, prevents the DHPG-induced inhibition of NMDAR-mediated mEPSCs *in vitro* [25,26], suggesting a preferential involvement of NMDARs over AMPARs in the learning and memory impairments induced by DHPG.

Many different mechanisms may underlie the initiation of NMDAR internalization induced by homologous (such as by NMDA/glycine) versus heterologous (such as by DHPG) stimulations [11,12,27,28,46,90]. PKD1 is activated following the regulated NMDAR endocytosis induced by either homologous or heterologous stimulations and thereby inhibits the activity of NMDARs remaining on cell surface [25,26]. As such, PKD1 activity can be regulated by multiple mechanisms including PKC phosphorylation [91], G-protein coupled receptors [92–94], P2Y(2) and P2X7 channels [95]. Moreover, stimulation of mGluR5 by application of DHPG in hippocampal cultures and slices results in the activation of PKD1, shown as an increase in phosphorylation of PKD1 at the site Ser-916 [45,94]. Further characterizing *in vivo* how intra-CA1 DHPG infusion affects NMDAR, AMPAR and PKD1 activities with regards to the learning and memory function will be of particular interest in light of the known association between alterations of NMDAR expression on the neuronal surface and the development of neuropsychological disorders and memory impairments [6–8,96].

## Supporting information

S1 Fig Correlation analysis between the swimming speed and the latency reaching the platform in the MWM task. The data measured from each non-operated (**A**), ACSF-infused (**B**) and DHPG-infused (**C**) rats at every day were respectively plotted in **A**, **B** and **C**. The left panels in **A**, **B** and **C** show data during training sessions at day 1 to day 5 (d1 –d5) before the infusion. The right panels show data during testing sessions for the second performance of MWM from the day 5 to day 7 (d5.1 –d7) in non-operated rats (**A**) or in rats after the CA1 infusion of ACSF (**B**) or DHPG (**C**). Pearson r: the correlation coefficient; n: the number of data collected. The linear regression is shown by the line through the data points.
(EPS)

S1 Table Statistical analysis conducted for data shown in Fig 2. % of distance: the percentages of swimming distance within the goal quadrant relative to the total swimming distance; % of time: the percentages of swimming time within the goal quadrant relative to the total swimming time; non: non-operated rats; before ACSF: before ACSF infusion; before DHPG: before DHPG infusion; after ACSF: after ACSF infusion; after DHPG: after DHPG infusion; /: compared with; d: the day consecutive from the first day of training in the MWM task.
(DOC)

S2 Table Statistical analysis conducted for data shown in Fig 3. % of distance: the percentages of swimming distance within the goal quadrant relative to the total swimming distance; % of time: the percentages of swimming time within the goal quadrant relative to the total swimming time.
(DOC)

S3 Table Statistical analysis conducted for data shown in Fig 4. % of distance: the percentages of swimming distance within the goal quadrant relative to the total swimming distance; % of time: the percentages of swimming time within the goal quadrant relative to the total swimming time; % of time in probe: the percentages of swimming time within the goal quadrant relative to the total swimming time in the probe test; Num. of times crossing: the number of times crossing the location of platform in the probe test. /: compared with; ACSF+ACSF: received ACSF following a pre-infusion of ACSF; ACSF+DHPG: receiving infusions of ACSF followed by DHPG; ACSF+CID: receiving infusions of ACSF followed by CID755673; MPEP+DHPG: receiving infusions of MPEP followed by DHPG; CID+DHPG: receiving infusions of CID755673 followed by DHPG. The MWM test was then performed 5 min after the second infusion.
(DOC)

S4 Table Statistical analysis conducted for data shown in Fig 5. After ACSF: after ACSF infusion; after DHPG: after DHPG infusion; after V(D): after the infusion of vehicle of Dynasore; after Dynasore: after Dynasore infusion; /: compared with; Object exploration in sample: the exploration of objects in the sample phase; Object exploration in test: The exploration of objects in the test phase;
(DOC)

S5 Table Statistical analysis conducted for data shown in Fig 6. Ctl RNA: rats received the intra-CA1 injection of control RNA; PKD1 siRNA: rats received the intra-CA1 injection of PKD1 siRNA; /: compared with; V(D): vehicle of Dynasore.
(DOC)
