# Supplementary material for: Hippocampal protein kinase D1 is necessary for DHPG-induced learning and memory impairments in rats
Source: PLoS One. 2018 Apr 3;13(4):e0195095. doi: 10.1371/journal.pone.0195095 (PMC5882104; doi:10.1371/journal.pone.0195095)
Supplement: S1 Table — % of distance: the percentages of swimming distance within the goal quadrant relative to the total swimming distance; % of time: the percentages of swimming time within the goal quadrant relative to the total swimming time; non: non-operated rats; before ACSF: before ACSF infusion; before DHPG: before DHPG infusion; after ACSF: after ACSF infusion; after DHPG: after DHPG infusion; /: compared with; d: the day consecutive from the first day of training in the MWM task. (DOC) [file pone.0195095.s002.doc]

**S1 Table. Statistical analysis conducted for data shown in Fig. 2**

|  | Comparisons | | Test methods | Test results |
| --- | --- | --- | --- | --- |
| Fig | Items | Rats |
| 2B | Swimming speed | non-operated | Repeated measures one-way ANOVA | F_4,28_ = 3.15, ***p* = 0.03** |
|  | Swimming speed | before ACSF | Repeated measures one-way ANOVA | F_4,36_ = 1.15, *p* = 0.35 |
|  | Swimming speed | before DHPG | Repeated measures one-way ANOVA | F_4,36_ = 1.23, *p* = 0.32 |
|  | Swimming speed | Non/before ACSF/before DHPG | Repeated measures two-way ANOVA | F_8,100_=0.43, *p* = 0.90 for interaction |
|  | Swimming speed | Before/after ACSF at d5 | paired *t*-tests | *t*_9_ = 0.25, *p* = 0.81 |
|  | Swimming speed | Before/after ACSF at d6 | paired *t*-tests | *t*_9_ = 0.20, *p* = 0.85 |
|  | Swimming speed | Before/after ACSF at d7 | paired *t*-tests | *t*_9_ = 0.04, *p* = 0.97 |
|  | Swimming speed | Before/after DHPG at d5 | paired *t*-tests | *t*_9_ = 0.99, *p* = 0.35 |
|  | Swimming speed | Non/after ACSF/after DHPG | Repeated measures two-way ANOVA | F_4,50_ = 0.05, *p* = 0.99 for interaction |
| 2C | Latency | non-operated | Repeated measures one-way ANOVA | F_4,28_ = 22.10, ***p* < 0.0001** |
|  | Latency | before ACSF | Repeated measures one-way ANOVA | F_4,36_ = 9.82, ***p* < 0.0001** |
|  | Latency | before DHPG | Repeated measures one-way ANOVA | F_4,36_ = 122.7, ***p* < 0.0001** |
|  | Latency | Non/before DHPG at d2 | Bonferroni post hoc test in two-way ANOVA | *t_16_* = 2.86, ***p* < 0.05** |
|  | Latency | Before/after DHPG at d5 | paired *t*-tests | *t*_9_ = 10.2, ***p* < 0.0001** |
|  | Latency | Non/After DHPG at d5 | Bonferroni post hoc test in two-way ANOVA | *t*_16_ = 3.70, ***p* < 0.01** |
|  | Latency | After DHPG/after ACSF at d5 | Bonferroni post hoc test in two-way ANOVA | *t*_16_ = 4.97, ***p* < 0.0001** |
| 2D | % of distance | non-operated | Repeated measures one-way ANOVA | F_4,28_ = 22.38, ***p* < 0.0001** |
|  | % of distance | before ACSF | Repeated measures one-way ANOVA | F_4,36_ = 70.94, ***p* < 0.0001** |
|  | % of distance | before DHPG | Repeated measures one-way ANOVA | F_4,36_ = 18.58, ***p* < 0.0001** |
|  | % of distance | Non/before ACSF at d2 | Bonferroni post hoc test in two-way ANOVA | *t_16_* = 3.04, ***p* < 0.05** |
|  | % of distance | Before/after ACSF at d5 | paired *t*-tests | *t*_9_ = 4.7, ***p* = 0.0011** |
|  | % of distance | Before/after ACSF at d6 | paired *t*-tests | *t*_9_ = 2.5, ***p* = 0.036** |
|  | % of distance | Before/after ACSF at d7 | paired *t*-tests | *t*_9_ = 2.8, ***p* = 0.021** |
|  | % of distance | Before/after DHPG at d5 | paired *t*-tests | *t*_9_ = 4.2, ***p* = 0.0025** |
|  | % of distance | Non/After DHPG at d5 | Bonferroni post hoc test in two-way ANOVA | *t*_16_ = 4.61, ***p* < 0.0001** |
|  | % of distance | After ACSF/after DHPG at d5 | Bonferroni post hoc test in two-way ANOVA | *t*_16_ = 5.33, ***p* < 0.0001** |
| 2E | % of time | non-operated | Repeated measures one-way ANOVA | F_4,28_ = 16.19, ***p* < 0.0058** |
|  | % of time | before ACSF | Repeated measures one-way ANOVA | F_4,36_ = 33.74, ***p* < 0.0001** |
|  | % of time | before DHPG | Repeated measures one-way ANOVA | F_4,36_ = 27.95, ***p* < 0.0001** |
|  | % of time | Non/before ACSF at d2 | Bonferroni post hoc test in two-way ANOVA | *t_16_* = 3.32, ***p* < 0.05** |
|  | % of time | Non/before ACSF at d3 | Bonferroni post hoc test in two-way ANOVA | *t_16_* = 3.41, ***p* < 0.01** |
|  | % of time | Before/after ACSF at d5 | paired *t*-tests | *t*_9_ = 0.63, *p* = 0.54 |
|  | % of time | Before/after ACSF at d6 | paired *t*-tests | *t*_9_ = 2.7, ***p* = 0.023** |
|  | % of time | Before/after ACSF at d7 | paired *t*-tests | *t*_9_ = 4.1, ***p* = 0.002** |
|  | % of time | Before/after DHPG at d5 | paired *t*-tests | *t*_9_ = 4.8, ***p* = 0.001** |
|  | % of time | Non/after DHPG at d5 | Bonferroni post hoc test in two-way ANOVA | *t*_16_ = 5.61, ***p* < 0.001** |
|  | % of time | After ACSF/after DHPG at d5 | Bonferroni post hoc test in two-way ANOVA | *t*_16_ = 4.35, ***p* < 0.001** |

% of distance: the percentages of swimming distance within the goal quadrant relative to the total swimming distance; % of time: the percentages of swimming time within the goal quadrant relative to the total swimming time; non: non-operated rats; before ACSF: before ACSF infusion; before DHPG: before DHPG infusion; after ACSF: after ACSF infusion; after DHPG: after DHPG infusion; /: compared with; d: the day consecutive from the first day of training in the MWM task.
