# Supplementary material for: Hippocampal protein kinase D1 is necessary for DHPG-induced learning and memory impairments in rats
Source: PLoS One. 2018 Apr 3;13(4):e0195095. doi: 10.1371/journal.pone.0195095 (PMC5882104; doi:10.1371/journal.pone.0195095)
Supplement: S2 Table — % of distance: the percentages of swimming distance within the goal quadrant relative to the total swimming distance; % of time: the percentages of swimming time within the goal quadrant relative to the total swimming time. (DOC) [file pone.0195095.s003.doc]

**S2 Table. Statistical analysis conducted for data shown in Fig 3**

|  | Comparisons | | Test methods | Test results |
| --- | --- | --- | --- | --- |
| Fig | Items | Rats |
| 3A | Swimming speed | Before/after DHPG at 100 µmol | paired *t*-tests | *t*_9_ = 1.5, *p* = 0.16 |
| 3B | Latency | Before/after DHPG at 0.5 µmol | paired *t*-tests | *t*_9_ = 2.58, ***p* = 0.028** |
|  | Latency | Before/after DHPG at 5 µmol | paired *t*-tests | *t*_9_ = 2.88, ***p* = 0.018** |
|  | Latency | Before/after DHPG at 50 µmol | paired *t*-tests | *t*_9_ = 10.19, ***p* < 0.0001** |
|  | Latency | Before/after DHPG at 100 µmol | paired *t*-tests | *t*_9_ = 4.12, ***p* = 0.0026** |
| 3C | % of distance | Before/after DHPG at 0.5 µmol | paired *t*-tests | *t*_9_ = 0.79, *p* = 0.45 |
|  | % of distance | Before/after DHPG at 5 µmol | paired *t*-tests | *t*_9_ = 4.07, ***p* = 0.0028** |
|  | % of distance | Before/after DHPG at 50 µmol | paired *t*-tests | *t*_9_ = 4.15, ***p* = 0.0025** |
|  | % of distance | Before/after DHPG at 100 µmol | paired *t*-tests | *t*_9_ = 7.18, ***p* < 0.0001** |
| 3D | % of time | Before/after DHPG at 0.5 µmol | paired *t*-tests | *t_9_* = 0.91, *p* = 0.39 |
|  | % of time | Before/after DHPG at 5 µmol | paired *t*-tests | *t_9_* = 2.32, ***p* = 0.045** |
|  | % of time | Before/after DHPG at 50 µmol | paired *t*-tests | *t_9_* = 4.23, ***p* = 0.0022** |
|  | % of time | Before/after DHPG at 100 µmol | paired *t*-tests | *t_9_* = 4.13, ***p* = 0.0025** |

% of distance: the percentages of swimming distance within the goal quadrant relative to the total swimming distance; % of time: the percentages of swimming time within the goal quadrant relative to the total swimming time.
