# Supplementary material for: Hippocampal protein kinase D1 is necessary for DHPG-induced learning and memory impairments in rats
Source: PLoS One. 2018 Apr 3;13(4):e0195095. doi: 10.1371/journal.pone.0195095 (PMC5882104; doi:10.1371/journal.pone.0195095)
Supplement: S3 Table — % of distance: the percentages of swimming distance within the goal quadrant relative to the total swimming distance; % of time: the percentages of swimming time within the goal quadrant relative to the total swimming time; % of time in probe: the percentages of swimming time within the goal quadrant relative to the total swimming time in the probe test; Num. of times crossing: the number of times crossing the location of platform in the probe test. /: compared with; ACSF+ACSF: received ACSF following a pre-infusion of ACSF; ACSF+DHPG: receiving infusions of ACSF followed by DHPG; ACSF+CID: receiving infusions of ACSF followed by CID755673; MPEP+DHPG: receiving infusions of MPEP followed by DHPG; CID+DHPG: receiving infusions of CID755673 followed by DHPG. The MWM test was then performed 5 min after the second infusion. (DOC) [file pone.0195095.s004.doc]

**S3 Table. Statistical analysis conducted for data shown in Fig 4**

|  | Comparisons | | Test methods | Test results |
| --- | --- | --- | --- | --- |
| Fig | Items | Rats |
| 4B | Latency | Before/after ACSF+DHPG | paired *t*-tests | *t*_9_ = 10.2, ***p* < 0.0001** |
|  | Latency | Before/after ACSF+ACSF | paired *t*-tests | *t*_9_ = 0.52, *p* = 0.62 |
|  | Latency | Before/after ACSF+MPEP | paired *t*-tests | *t*_9_ = 1.1, *p* = 0.29 |
|  | Latency | Before/after ACSF+CID | paired *t*-tests | *t*_9_ = 0.02, *p* = 0.98 |
|  | Latency | Before/after MPEP+DHPG | paired *t*-tests | *t*_9_ = 0.53, *p* = 0.61 |
|  | Latency | Before/after CID+DHPG | paired *t*-tests | *t*_9_ = 0.12, *p* = 0.90 |
| 4C | % of distance | Before/after ACSF+DHPG | paired *t*-tests | *t*_9_ = 4.5, ***p* = 0.0016** |
|  | % of distance | Before/after ACSF+ACSF | paired *t*-tests | *t*_9_ = 1.1, *p* = 0.29 |
|  | % of distance | Before/after ACSF+MPEP | paired *t*-tests | *t*_9_ = 0.03, *p* = 0.98 |
|  | % of distance | Before/after ACSF+CID | paired *t*-tests | *t*_9_ = 0.11, *p* = 0.91 |
|  | % of distance | Before/after MPEP+DHPG | paired *t*-tests | *t*_9_ = 1.0, *p* = 0.33 |
|  | % of distance | Before/after CID+DHPG | paired *t*-tests | *t*_9_ = 0.38, *p* = 0.71 |
| 4D | % of time | Before/after ACSF+DHPG | paired *t*-tests | *t*_9_ = 4.2, ***p* = 0.0022** |
|  | % of time | Before/after ACSF+ACSF | paired *t*-tests | *t*_9_ = 0.18, *p* = 0.86 |
|  | % of time | Before/after ACSF+MPEP | paired *t*-tests | *t*_9_ = 0.86, *p* = 0.41 |
|  | % of time | Before/after ACSF+CID | paired *t*-tests | *t*_9_ = 0.0003, *p* = 0.99 |
|  | % of time | Before/after MPEP+DHPG | paired *t*-tests | *t*_9_ = 0.54, *p* = 0.61 |
|  | % of time | Before/after CID+DHPG | paired *t*-tests | *t*_9_ = 0.37, *p* = 0.72 |
| 4E | % of time in probe | After ACSF+ACSF/after ACSF+DHPG | unpaired *t*-tests | *t*_18_ = 3.194, ***p* = 0.005** |
|  | % of time in probe | After ACSF+ACSF/after ACSF+MPEP | unpaired *t*-tests | *t*_18_ = 0.459, *p* = 0.652 |
|  | % of time in probe | After ACSF+ACSF/after ACSF+CID | unpaired *t*-tests | *t*_18_ = 0.017, *p* = 0.987 |
|  | % of time in probe | After ACSF+ACSF/after MPEP+DHPG | unpaired *t*-tests | *t*_18_ = 0.729, *p* = 0.475 |
|  | % of time in probe | After ACSF+ACSF/after CID+DHPG | unpaired *t*-tests | *t*_18_ = 0.415, *p* = 0.683 |
| 4F | Num. of times crossing | After ACSF+ACSF/after ACSF+DHPG | unpaired *t*-tests | *t*_18_ = 4.38, ***p* = 0.0004** |
|  | Num. of times crossing | After ACSF+ACSF/after ACSF+MPEP | unpaired *t*-tests | *t*_18_ = 1.74, *p* = 0.864 |
|  | Num. of times crossing | After ACSF+ACSF/after ACSF+CID | unpaired *t*-tests | *t*_18_ = 1.77, *p* = 0.861 |
|  | Num. of times crossing | After ACSF+ACSF/after MPEP+DHPG | unpaired *t*-tests | *t*_18_ = 1.10, *p* = 0.286 |
|  | Num. of times crossing | After ACSF+ACSF/after CID+DHPG | unpaired *t*-tests | *t*_18_ = 1.12, *p* = 0.277 |

% of distance: the percentages of swimming distance within the goal quadrant relative to the total swimming distance; % of time: the percentages of swimming time within the goal quadrant relative to the total swimming time; % of time in probe: the percentages of swimming time within the goal quadrant relative to the total swimming time in the probe test; Num. of times crossing: the number of times crossing the location of platform in the probe test. /: compared with; ACSF+ACSF: received ACSF following a pre-infusion of ACSF; ACSF+DHPG: receiving infusions of ACSF followed by DHPG; ACSF+CID: receiving infusions of ACSF followed by CID755673; MPEP+DHPG: receiving infusions of MPEP followed by DHPG; CID+DHPG: receiving infusions of CID755673 followed by DHPG. The MWM test was then performed 5 min after the second infusion.
