# Supplementary material for: Hippocampal protein kinase D1 is necessary for DHPG-induced learning and memory impairments in rats
Source: PLoS One. 2018 Apr 3;13(4):e0195095. doi: 10.1371/journal.pone.0195095 (PMC5882104; doi:10.1371/journal.pone.0195095)
Supplement: S4 Table — After ACSF: after ACSF infusion; after DHPG: after DHPG infusion; after V(D): after the infusion of vehicle of Dynasore; after Dynasore: after Dynasore infusion; /: compared with; Object exploration in sample: the exploration of objects in the sample phase; Object exploration in test: The exploration of objects in the test phase; (DOC) [file pone.0195095.s005.doc]

**S4 Table. Statistical analysis conducted for data shown in Fig 5**

|  | Comparisons | | Test methods | Test results |
| --- | --- | --- | --- | --- |
| Fig | Items | Rats |
| 5A | total distance moved | After ACSF/after DHPG | two-way ANOVA | F_1,34_ < 1, *p* = 0.999 |
|  | total distance moved | After V(D)/after Dynasore | two-way ANOVA | F_1,34_ < 1, *p* = 0.882 |
|  | total distance moved | After DHPG/after Dynasore | two-way ANOVA | F_1,34_ = 1.58, *p* = 0.214 for interaction |
| 5B | Moving speed | After ACSF/after DHPG | two-way ANOVA | F_1,34_ = 0, *p* = 0.999 |
|  | Moving speed | After V(D)/after Dynasore | two-way ANOVA | F_1,34_ < 1, *p* = 0.890 |
|  | Moving speed | After DHPG/after Dynasore | two-way ANOVA | F_1,34_ = 1.62,  *p* = 0.212 for interaction |
| 5C | Time spent in the center | After ACSF/after DHPG | two-way ANOVA | F_1,34_ < 1, *p* = 0.784 |
|  | Time spent in the center | After V(D)/after Dynasore | two-way ANOVA | F_1,34_ = 1.02, *p* = 0.320 |
|  | Time spent in the center | After DHPG/after Dynasore | two-way ANOVA | F_1,34_ = 2.08,  *p* = 0.158 for interaction |
| 5D | Time spent in corners | After ACSF/after DHPG | two-way ANOVA | F_1,34_ < 1, *p* = 0.833 |
|  | Time spent in corners | After V(D)/after Dynasore | two-way ANOVA | F_1,34_ = 1.69, *p* = 0.202 |
|  | Time spent in corners | After DHPG/after Dynasore | two-way ANOVA | F_1,34_ = 4.27, ***p* = 0.046 for** **interaction** |
| 5E | Object exploration in sample | After ACSF/after DHPG | two-way ANOVA | F_1,34_ < 1, *p* = 0.556 |
|  | Object exploration in sample | After V(D)/after Dynasore | two-way ANOVA | F_1,34_ < 1, *p* = 0.798 |
|  | Object exploration in sample | After DHPG/after Dynasore | two-way ANOVA | F_1,34_ < 1,  *p* = 0.588 for interaction |
| 5F | Object exploration in test | After ACSF/after DHPG | two-way ANOVA | F_1,34_ < 1, *p* = 0.921 |
|  | Object exploration in test | After V(D)/after Dynasore | two-way ANOVA | F_1,34_ < 1, *p* = 0.510 |
|  | Object exploration in test | After DHPG/after Dynasore | two-way ANOVA | F_1,34_ < 1,  *p* = 0.739 for interaction |

After ACSF: after ACSF infusion; after DHPG: after DHPG infusion; after V(D): after the infusion of vehicle of Dynasore; after Dynasore: after Dynasore infusion; /: compared with; Object exploration in sample: the exploration of objects in the sample phase; Object exploration in test: The exploration of objects in the test phase;
