# Supplementary material for: Hippocampal protein kinase D1 is necessary for DHPG-induced learning and memory impairments in rats
Source: PLoS One. 2018 Apr 3;13(4):e0195095. doi: 10.1371/journal.pone.0195095 (PMC5882104; doi:10.1371/journal.pone.0195095)
Supplement: S5 Table — Ctl RNA: rats received the intra-CA1 injection of control RNA; PKD1 siRNA: rats received the intra-CA1 injection of PKD1 siRNA; /: compared with; V(D): vehicle of Dynasore. (DOC) [file pone.0195095.s006.doc]

S5 Table. Statistical analysis conducted for data shown in Fig 6

|  | Comparisons | | Test methods | Test results |
| --- | --- | --- | --- | --- |
| Fig | Items | Rats |
| 6A | % of exploration time in control | After ACSF | one-sample *t*-test vs. 50% | *t*_6_ = 2.15, ***p* = 0.037** |
|  | % of exploration time in control | After DHPG | one-sample *t*-test vs. 50% | *t*_6_ = -2.19. *p* = 0.965 |
|  | % of exploration time in control | After ACSF/after DHPG | Unpaired *t*-test | *t*_12_ = 2.88, ***p* = 0.014** |
|  | % of exploration time in V (D) | After ACSF | one-sample *t*-test vs. 50% | *t*_8_ = 1.95, ***p* = 0.043** |
|  | % of exploration time in V (D) | After DHPG | one-sample *t*-test vs. 50% | *t*_8_ = -1.69, *p* = 0.935 |
|  | % of exploration time in V (D) | After ACSF/after DHPG | Unpaired *t*-test | *t*_16_ = 2.55, ***p* = 0.021** |
|  | % of exploration time in Dynasore | After ACSF | one-sample *t*-test vs. 50% | *t*_8_ = 1.91, ***p* = 0.044** |
|  | % of exploration time in Dynasore | After DHPG | one-sample *t*-test vs. 50% | *t*_9_ = 1.71, *p* = 0.061 |
|  | % of exploration time in Dynasore | After ACSF/after DHPG | Unpaired *t*-test | *t*_18_ = 0.22, *p* = 0.829 |
| 6B | PKD1 expression | Ctl RNA/PKD1 siRNA | Unpaired *t*-test | *t*_13_ = 2.33, ***p* = 0.036** |
| 6C | % of exploration time in ctl RNA | After ACSF | one-sample *t*-test vs. 50% | *t*_6_ = 0.70, *p* = 0.254 |
|  | % of exploration time in ctl RNA | After DHPG | one-sample *t*-test vs. 50% | *t*_8_ = -3.40, *p* = 0.995 |
|  | % of exploration time in ctl RNA | After ACSF/after DHPG | Unpaired *t*-test | *t*_14_ = 2.26, ***p* = 0.040** |
|  | % of exploration time in PKD1 siRNA | After ACSF | one-sample *t*-test vs. 50% | *t_6_* = 3.05, ***p* = 0.014** |
|  | % of exploration time in PKD1 siRNA | After DHPG | one-sample *t*-test vs. 50% | *t*_8_ = 2.32, ***p* = 0.024** |
|  | % of exploration time in PKD1 siRNA | After ACSF/after DHPG | Unpaired *t*-test | *t*_13_ = 0.62, *p* = 0.543 |

Ctl RNA: rats received the intra-CA1 injection of control RNA; PKD1 siRNA: rats received the intra-CA1 injection of PKD1 siRNA; /: compared with; V(D): vehicle of Dynasore
